# Nutritional deficiencies in homeless persons with problematic drinking: a systematic review

**DOI:** 10.1186/s12939-017-0564-4

**Published:** 2017-05-05

**Authors:** Sharea Ijaz, Joni Jackson, Helen Thorley, Katie Porter, Clare Fleming, Alison Richards, Adrian Bonner, Jelena Savović

**Affiliations:** 1 The National Institute for Health Research Collaboration for Leadership in Applied Health Research and Care West (NIHR CLAHRC West), University Hospitals Bristol NHS Foundation Trust, 9th Floor Whitefriars, Lewins Mead, Bristol, BS1 2NT UK; 2 School of Social and Community Medicine, University of Bristol, Bristol, UK; 3 Bristol City Council, St Annes House, Bristol, BS4 4AB UK; 4 Compass Health, The Compass Centre, 1 Jamaica Street, Bristol, BS2 8JP UK; 5 Research and Development Unit, The Salvation Army, London, SE1 6BN UK

**Keywords:** Homeless, Alcohol, Systematic review, Problem drinking, Malnutrition

## Abstract

**Background:**

A significant proportion of homeless people drink alcohol excessively and this can lead to malnutrition and consequent medical problems. The aim of this review was to assess the evidence on the range of nutritional deficiencies in the homeless problem-drinking populations.

**Methods:**

We conducted a comprehensive search of nine scientific literature databases and 13 grey literature sources. We included studies of any design that included homeless population with problem-drinking and reported measures of nutritional deficiencies in urine or blood. Study selection and data extraction was done by one reviewer and checked by another. Data on malnutrition profile were summarized narratively.

**Results:**

We found nine studies reporting nutritional deficiencies in homeless populations with problem-drinking. The oldest study was from the 1950s and the most recent from 2013. The following nutrients were reported across studies: vitamins B1, B2, B6, B9, B12, C, A, and E; haemoglobin; and albumin. The most common deficiencies reported were of vitamin B1 (prevalence of deficiency was 0, 2, 6, 45, and 51% in five studies) and vitamin C (29, 84, and 95% in three studies). None of the studies were assessed to be at a low risk of bias.

**Conclusions:**

The limited, low quality and relatively old evidence suggests that homeless people who drink heavily may be deficient in vitamin C, thiamine, and other nutrients. New, well conducted studies are needed in order to optimally inform public health interventions aimed at improving deficiencies in this population.

**Trial Registration:**

PROSPERO CRD42015024247

**Electronic supplementary material:**

The online version of this article (doi:10.1186/s12939-017-0564-4) contains supplementary material, which is available to authorized users.

## Background

**Fig. 1:**
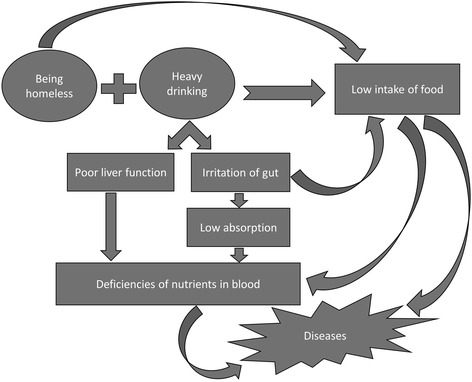
Nutritional deficiency mechanisms in Homeless problem-drinkers

Nearly two thirds of homeless people in the UK drink more alcohol than is recommended [1]. The impact of homelessness on an individual, combined with the effects of excessive drinking can become a double blow on nutritional health and lead to deficiencies [2] (Fig. 1). These deficiencies contribute to neurological and other organ damage and can lead to long-lasting medical conditions [3].

Historically vitamin B1 (thiamine) deficiency has been associated with homeless and problem-drinking populations and the observed neurological damage in these groups is attributed to its deficiency. Parenteral thiamine is therefore recommended for patients with suspected chronic alcohol misuse who are admitted to accident and emergency (A&E) departments to prevent Wernicke’s encephalopathy [4]. However, to our knowledge no comprehensive collation of evidence on the prevalence or severity of vitamin deficiencies in this population has been done recently. Knowing the range of the most common nutritional deficiencies can help develop an intervention to counter these deficiencies which can prevent illness in this high-risk group. In the long term, this could save costs for the health system, especially in A&E attendances [5], as well as improve the health and quality of life of the homeless and the vulnerably housed people who consume alcohol excessively. This systematic review of literature was undertaken alongside a complementary systematic review of effectiveness of interventions for improving or preventing malnutrition in this population [6]. The aim of this review is to assess the evidence on prevalence and range of nutritional deficiencies in the homeless problem-drinking population. The findings of both reviews will inform the development of an intervention for nutritional deficiencies (e.g. supplements or fortified food products).

## Methods

### Literature searches

A single, comprehensive search strategy was developed and applied to multiple databases to find all relevant nutritional literature on homeless problem drinkers. This search strategy was used to identify studies, both for the current review of prevalence of malnutrition and the complementary review of intervention studies. The search was published in the protocol [6] and is up to date until 16th November 2016.

### Study inclusion and exclusion criteria

We included studies of any design reporting nutrient levels in the homeless problem-drinking population. We included studies that fit the legal definition of homelessness used in the UK, which includes the following: sleeping rough (outside); residing in temporary accommodation such as hostels, bed and breakfasts, or night shelters; staying on a temporary basis with family or friends (‘sofa surfers’); currently housed people who are at risk of being evicted; and currently housed people who cannot stay either: because they cannot afford to stay, or the home is in a very poor condition, or they are subject to violence, abuse or threats in the home [7]. Studies with participants of any age or gender were included as long as the majority of the population contained both problem-drinkers and homeless.

To be eligible studies had to report urinary or blood levels of any of the following nutrients: Vitamin A (Retinol, retinal, beta carotene), B1 (thiamine); B2 (riboflavin); B3 (niacin or nicotinic acid); B5 (pantothenic acid); B6 (pyridoxine, pyridoxal, pyridoxamine); B7 (biotin); B9 (folic acid), B12 (cobalamins), C (ascorbic acid), D (cholecalciferol/D3, ergocalciferol/D2), E (tocopherols, tocotrienols), K (phylloquinone, menaquinones), haemoglobin, albumin, ferritin, or iron.

We excluded the following: position papers; editorials; commentaries; studies where the participants were either homed alcohol drinkers or homeless without drinking problems; institutionalised people; studies where entire communities are homeless (e.g. occupiers of slums or shanty towns, refugees or similar populations); people homeless due to mass displacement (related to war, famine or natural disaster); non-nutrient outcomes (e.g. weight or BMI, muscle or fat mass); and studies of dietary intake (fruit/vegetables/fortified food/vitamins) as these are subject to recall bias and overlook the deficiency related to poor absorption or liver function – the consequences of problem-drinking.

At the screening stage [6], two reviewers independently tagged studies that were potentially relevant to the current and the intervention review. These tagged records were screened again by a third reviewer for relevance. In addition, the third reviewer independently screened the remaining untagged studies to ensure relevant papers were not missed. References of included primary studies as well as identified systematic reviews were searched to identify any additional studies. Identified relevant studies were assessed in full text for inclusion by one reviewer and the decision checked by another, with any disagreements resolved by a third reviewer.

### Data extraction strategy

Data extraction was carried out by one reviewer on a pre-specified pro-forma in Microsoft Excel and checked by another. Disagreements were resolved by discussion with a third reviewer. Data were extracted for the following: author, year of publication, report type, year(s) of data collection, location of study, study design, aim, population, sample size, proportion of problem-drinkers and homeless in sample, ethnicity, gender, age, type of homelessness (all/shelter/rough sleeper etc.), deficiency measures and definitions reported in the study, method used to measure deficiency level, deficiencies of vitamins as reported (mean/SD; proportion deficient), and any other deficiency measures/outcomes reported.

### Risk of bias assessment

There is no single widely accepted risk of bias tool for cross sectional or survey type studies of prevalence. We therefore considered the previous work in this area [8–11] and adopted some of the key criteria described in these studies. All these assessed three key areas of potential bias in prevalence studies: a representative and appropriately recruited sample, a valid and reliable measurement of the condition under study, and whether the analysis was adjusted for important covariates. We considered the following factors to be important confounding factors: age, sex, duration of homelessness/alcohol problems, and assessed these three areas of bias in our included studies. The overall risk of bias for a study was considered ‘low’ when the following three conditions were met: (i) all criteria related to the representativeness of study sample were judged to be at low risk of bias; (ii) at least 2 criteria related to measurement of deficiencies were judged to be at low risk; and (iii) the analysis was adjusted for important confounders.

### Data synthesis and presentation

Data were tabulated for each nutrient. The included studies were very heterogeneous with respect to their population and time period so it was not considered appropriate to conduct formal meta-analyses and produce quantitative summary estimates of deficiencies across studies. Instead, we carried out a narrative synthesis. We did however produce forest plots without summary estimates to aid the presentation of the results (See Additional files 1 and 2).

## Results

**Fig. 2:**
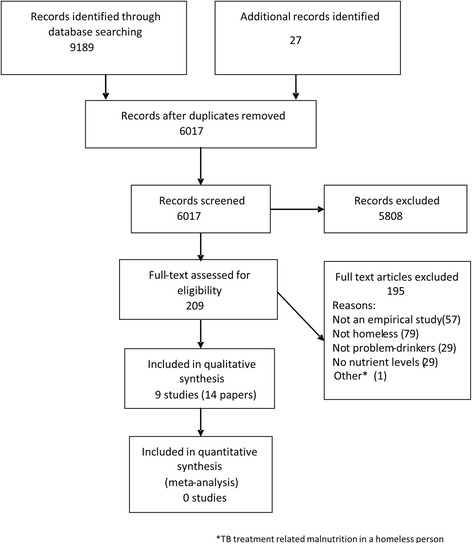
PRISMA flow diagram for review process

The search identified 6017 references. Of these 190 were initially tagged as potentially relevant for the current review and 19 further records were identified through rescreening. A total of 209 references were identified for full paper assessment. Of these, 14 papers (referring to nine studies) were included and 195 papers were excluded (Fig. 2). The online supplement contains the list of all excluded studies (Additional file 1).

Six of the included studies (reported in 11 papers) were surveys [12–22], two were case reports [23, 24], and one was an intervention study [16].

Most studies were small. The median number of participants included in studies was 39 (IQR 8–71). The total number of participants included in this review is 621. Three studies [17, 22, 24] included ethnic Black participants while others provided no information on ethnicity. In the four studies that included women, they comprised a small proportion of the sample (between 6 and 13%). Age of the participants was more or less the same across the studies, ranging from 19 to 76 years with an average of 46 years (IQR 43.25–48.75). Participants recruited were either from the streets (rough sleepers) [17, 18, 21, 23, 24], or from shelters and clinics [13, 16, 18, 22]. When reported, [18, 21], the duration of homelessness varied greatly, ranging between one month to 55 years among the participants. Table 1 displays key characteristics of included studies.

**Table 1:** Key characteristics of included studies

First author	Publication year	Year of data collection	Country	Study design	Source population	Sample analysed	Type of homelessness	Problem-drinkers % in the sample	Ethnicity	Gender M%	Age years mean (range)
Figueroa	1953	1949	USA	Survey	Alcoholic homeless men in a Chicago prison	24	Rough sleepers	100%	8.3% Black	100%	45 (20–60+)
Darnton-Hill	1986	1981–86	Australia	Survey	Men from two Sydney hostels and one clinic for homeless	39^a^	Hostel	70%	NR	100%	51.5 (26–76)
van der Westhuyzen	1987	NR	South Africa	Comparative survey	Men from 24 homeless hostels in Pretoria	49	Hostel	100%	Black	100%	38 (22–65)
Drijver	1993	NR	Netherlands	Intervention study	Rotterdam homeless houses	10	Hostel	100%	NR	90%	48 (38–64)
Kertesz	2001	1998	USA	Case report	NR	2	Rough sleepers	100%	one black person, other NR	100%	(55–58)
Malmauret	2002	1999–2000	France	Survey	Adult rough sleepers in Paris	71	Rough sleepers	84%	NR	88.5%	48 (26–76)
Fung	2005	NR	Australia	Case report	A homeless person seeking medical help	1	Rough sleeper	100%	NR	100%	44
Kubisova	2008	2003	Czech Republic	Survey	homeless people on streets in Prague	201	Rough sleepers; hostel	Likely > 50%	NR	87% M	41 (19–70)
Lee	2014	2013	South Korea	Retrospective survey	homeless visiting emergency department	217	NR	100%	NR	96% M	51 (44–56)

*NR* not reported, *M* male

^a^Refers to the subgroup (of total 107 men) that were not taking vitamin supplements

### Risk of bias in included studies

**Fig. 3:**
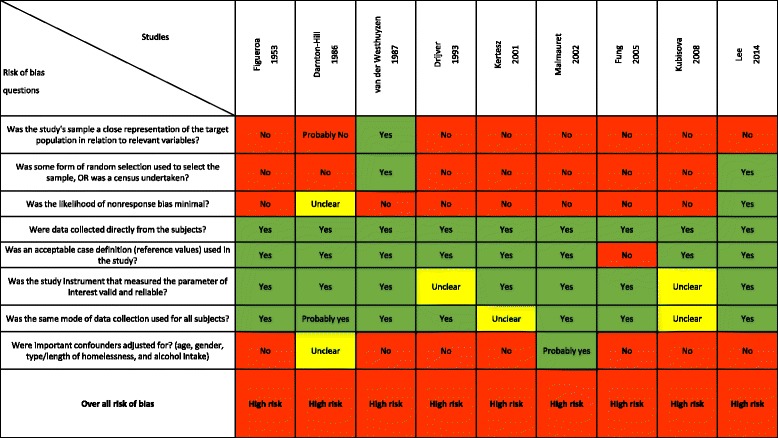
Risk of bias across included studies

None of the included studies were at low risk of bias (Fig. 3). The risk of bias related to the representativeness of the sample was high in all but one study. The vitamin levels in the blood were usually assessed using valid and reliable methods across study participants. However, for most studies the analysis did not adjust for important covariates (age, sex, duration of homelessness/alcohol problems).

### Data synthesis

#### Deficiencies reported

The most commonly reported data were vitamin B1 levels (6 studies [13, 16, 17, 19, 21, 22] followed by B6, and B12 (5 studies [13, 16, 19, 21, 22]). Vitamin C, B9, and haemoglobin levels were reported in three studies, while vitamin A, B2, and E levels were reported in a single study each.. Vitamins B3, B5, B7, D, and K levels were not reported in any included studies.

The measurement methods were variable across studies. This probably reflects the advancement of laboratory techniques for assessment of nutrients over the past several decades. For example, the oldest study measured fasting excretion level in urine for the vitamins B1 and B2 and found that none of the participants were deficient in thiamine. In three later studies, vitamin B1 deficiency was measured using various measures in blood: the thiamine pyrophosphate (TTP) effect [13], the erythrocyte thiamine level [22], and the thiamine diphosphate (TDP) effect [16] and transketolase activity (TK activity) [13] and these studies used varied techniques and instruments for these measurements (Table 2).

**Table 2:** Measurement of deficiencies in the included studies

Author/Year	Deficiency/measure reported	Definition of deficiency	Technique used to measure
Figueroa 1953 [17]	Fasting Hour Excretion of B1 and B2	B1 = < 0.6 microgram./hr; B2 = 20 μ/hr	Photofluorometery: B1 = Hennesey and Cerecedo’s technique; B2 = Connor and Straub’s method.
Darnton-Hill 1986 [13]	TPP effect and TK activity for B1; blood levels of: B6; B9; B12; C; albumin a	TPP effect for B1= > 14% activity; TK activity for B1 = < 130 μmol/min/L; B6 = < 300 μmol/min/L;P5P for B6= > 50% activity; B12 = <150pmol/L; Iron = <10 μmol/L; C = < 23 μmol/L; serum B9 = <4 ng/ml	B1 = measuring colorimetrically the formation of sedoheptulose-7-phosphate with ribose-5- phosphate as the substrate; B6 = erythrocyte transaminase and pryidoxal5 phosphate activation. B9 and B12 = isotopic radioassay; C = colorimetric procedure using dinitrophenylhydrazine.
van der Westhuyzen 1987 [22]	Blood levels of: B6; B9; B12; albumin; erythrocyte level of B1	Reference ranges determined in healthy subjects: B6 = 26–96 nmol/; B1 = 50–106 microgram/liter; B12 = 160–900 ng/l; serum folate = 2–13microgram/liter; serum I'-glutamyltransferase (GGT) = 10–50 U/I.	Radio-assay kit; Biochromatic Analyzer commercial kits; For B1 = automated microbiological assay using a streptomycin-resistant mutant of Lactobacillus fermenci as the test organism.
Drijver 1993 [16]	TDP effect and TK activity for B1; Blood levels of: B6; B12	TDP effect reference range = 0–25%; TK activity reference range = 10.4–15.1 U/mmol Hb; PLP effect reference range = 35–107 nmol/l;	HPLC; PLP measured with a fluorimeter
Kertesz 2001 [24]	Levels NR. Results of the following were found normal: B12; B 9; iron	NR	Hospital tests
Malmauret 2002 [21]	Blood levels of vitamins: A; B1; B6; B9; B12; C; E	B6 = 23–100 nmol/l; B12 = 160–420 pmol/l; B1 = 6–40 mmol/l.	HPLC and radioimmunoassay
Fung 2005 [23]	Anaemia, albumin level	NR	NR
Kubisova 2008 [18]	Blood levels of: prealbumin; albumin; haemoglobin	Albumin <33 g/l; prealbumin <0.16 g/l.	NR
Lee 2014 [20]	Blood levels of: B1; B6; B12; C; haemoglobin	Reference ranges for vitamins: B1 = 59 to 213; B12 = 200 to 950; B6 = 20 to 202; C = 26.1 to 84.6	Vitamin B1, B6, and C = HPLC; B12 levels = electro-chemiluminescence immunoassay

*A* Retinol/beta carotene, *B1* thiamine, *B2* riboflavin, *B3* niacin, *B5* pantothenic acid, *B6* pyridoxine, *B7* biotin, *B9* folic acid, *B12* cobalamins, *C* ascorbic acid, *D* ergo/cholecalciferol, *E* tocopherols, *Hb* haemoglobin, *HPLC* high performance liquid chromatography, *NR* not reported, *P5P* pyridoxal 5 phosphate, *PLP* pyridoxal 5 phosphate, *TDP* thiamine diphosphate, *TK* transketolase, *TPP* thiamine pyrophosphate

The definitions or thresholds for deficiencies also varied. For example, for thiamine the following five definitions were used across studies: (i) excretion of less than 0.6 microgram/hr thiamine; (ii) thiamine pyrophosphate (TPP) effect for thiamine deficiency in range of 15 to 44 where 0–14 is adequate; (iii) thiamine diphosphate (TDP)-effect for thiamine deficiency where reference range is 0–25%; (iv) reference range from 59 to 213 mmol/l; and (v) reference range from 50 to 106 microgram/litre. All the studies reported deficiencies as average (mean/median) levels for groups, with or without measures of spread (SD, IQR). Six studies also reported the proportion of people found deficient based on reference values for the general population.

#### Evidence on deficiencies- Prevalence and severity

The range of mean circulating vitamin levels observed across the included studies is presented in Table 3. No clear pattern of nutrient deficiency was observed across the studies for any of the vitamins. The mean levels of vitamins varied widely across the studies. For studies which reported data on the proportion of participants below deficiency thresholds, the most common deficiency was of vitamin C. All three studies that measured this factor found a significant proportion (29, 84, and 95%) of participants were deficient. The other common deficiency was for vitamin B1, with two studies from the 1980s reporting nearly half of the studied population to be deficient. However, the two more recent studies [19, 21] found just 2% and 6% were deficient, respectively. For vitamins B2, B6, B9, B12 and vitamin E the prevalence of deficiencies was low (0 to 23%). In the single study assessing vitamin A 44% of the participants were found to be deficient [21].

**Table 3:** Nutritional deficiencies in homeless problem-drinkers reported in the included studies

Study	Number	Deficient proportion (%) of the participants										Deficiency levels - Mean ± SD (range)									
		B1	B 2	B6	B9	B12	C	A	E	Hb	Albumin	B1	B 2 μ/hr	B6	B9	B12	C μmol/L	A μmol/L	E μmol/L	Hb g/dL	Albumin g/L
Figueroa 1953 [17]	24	0	18	.	.	.	.	.	.	9.2	.	2.9 μ/hr	70	.	.	.	.	.	.	15.2	.
Darnton-Hill 1986 [13]	39	45	.	20	31	0	29	.	.	.	26	TPP 15.3 ± 10.5	.	P5P57.0 ± 26.6	3.6 ± 4.0 ng/ml	341 ± 203 ng/ml	34.9 ± 16.2	.	.	.	.
van der Westhuyzen 1987 [22]	49	51.2	.	7.3	4.1	0	.	.	.	.	0	51.5 ± 11.0 μg/L	.	61.9 ± 25.1 nmol/L	4.3 ± 1.8 (R 1.0–9.0) ng/ml	0.648 ± 0.235 ng/ml	.	.	.	.	38.9 ± 2.5
Drijver 1993 [16]	10	.	.	.	.	.	.	.	.	.	.	TDP 18	.	PLP 34.2 nmol/L	.	TK 9.6 μ/mmolHb	.	.	.	.	.
Kertesz 2001 [24]	1	.	.	.	.	.	.	.	.	.	.	.	.	.	.	.	.	.	.	.	35 ± 2.5
Malmauret 2002 [21]	71	5.6	.	5.6	0	14.1	95	43.6	19.7	.	.	58 ± 104 μmol/L	.	152 ± 143 nmol/L	12.7 ± 7.3 nmol/L	385 ± 225 pmol/L	16 ± 8	1.88 ± 0.95	22 ± 7	.	.
Fung 2005 [23]	1	.	.	.	.	.	.	.	.	100	.	.	.		.		.	.	.	10.8	28
Kubisova 2008 [18]	201	.	.	.	.	.	.	.	.	.	2	.	.		.		.	.	.	14 ± 0.1 (M); 12.1 ± 0.2 (F)	42 ± 4
Lee 2014 [20]	217	2.3	.	23.5	.	2.3	84.3	.	.	.	.	145.8 (108.5–197.7) nmol/L	.	34.2 (20.5–65.45) nmol/L	.	0.617 (0.458–0.918) ng/ml	11.60 (3.65–21.55)	.	.	14 IQR (12.4–15.3)	.

*A* Retinol/beta carotene, *B1* thiamine, *B2* riboflavin, *B3* niacin, *B5* pantothenic acid, *B6* pyridoxine, *B7* biotin, *B9* folic acid, *B12* cobalamins, *C* ascorbic acid, *D* ergo/cholecalciferol, *E* tocopherols, *Hb* haemoglobin, *HPLC* high performance liquid chromatography, *F* female, *IQR* interquartile range, *M* male, *N* number of participants, *NR* not reported, *P5P* pyridoxal 5 phosphate effect, *PLP* pyridoxal 5 phosphate effect, *R* range, *TDP* thiamine diphosphate, *TK* transketolase activity, *TPP* thiamine pyrophosphate effect

Kertesz 2001 [24] reported two cases of clinically diagnosed pellagra but did not measure niacin (B3) levels. Both patients had avoided meals and shelter for several months and relied largely on alcohol for sustenance and both recovered with food and niacin supplements. The single case in Fung 2005 had also lived on several litres of wine a day for a year. Vitamin status was not checked in this person but the patient was found to be anaemic. The proportion with anaemia was reported in one other study [17]. Mean haemoglobin levels were reported in three other studies, all within normal range, with only one study reporting data separately for women and men [18]. Hypoalbuminemia (deficient serum albumin) was reported in three studies with the proportion ranging from 0 to 26%.

We did not observe any obvious differences in nutritional deficiencies between different subgroups, e.g. in rough sleepers vs hostel dwellers. Similarly, since only single studies were identified from most locations, no clear difference was seen by country. We also presented graphically the prevalence of deficiencies for all vitamins, as reported in individual studies, to illustrate variation across populations and time (See Additional file 2).

## Discussion

### Summary of findings

We found that nutritional deficiencies have been observed to a variable extent in the homeless problem-drinking populations. Traditionally, vitamin B1 deficiency has been associated with this population and this is consistent with the findings of the two studies from the 1980s. However, in more recent years vitamin C deficiency appears to be more common [13, 19, 21]. The evidence however is of low quality, heterogeneous, and based on few studies, with only one [19] from this decade. This precludes effective use of these data to inform current clinical or public health practice. In addition, no studies measured vitamins B3, B5, B7, D, and K. Considering the evidence on global vitamin D deficiency [25] and its recommended supplementation [26] it is possible that the need for its supplementation also exists in homeless drinkers. The single study measuring vitamin A and E found a significant minority of the participants below reference levels [21].

### Comparison with previous work

To our knowledge this is the first systematic review that evaluated deficiencies in this specific population. Our search was not limited by date or language allowing a comprehensive assessment of research across time and locations. Additionally, an extensive grey literature search ensured that we captured all relevant research pertaining to this population.

One previous systematic review [27] reported on the nutrition profile of the adult homeless in Paris. This review, based on a single study [21], found vitamin C to be an important deficiency for the homeless, in line with our findings. The majority of traditional reviews on alcohol-related nutrient deficiencies considered problem-drinking in the general population alone, and not in the homeless subgroup. All of these recommended further studies with better assessment of deficiencies [28–32]. Two reviews that have addressed the homeless subgroup of problem drinkers [2, 33] also concluded that better assessment of their nutritional status was needed.

### Limitations

The full range and severity of deficiencies may differ from those we found. This is partly because diets have changed over time in the general population [34, 35] and homeless heavy drinkers today are probably eating differently from those in the previous century. Also, the studies almost always included a convenient, rather than representative sample. Thus the proportion of homeless with a deficiency may be significantly higher (or lower) than that seen in the included studies.

The studies were few and varied. None of the included studies were at low risk of bias. The study with least risk of bias was the most recent study, from South Korea [19]. It is also important to consider applicability (external validity) of study findings to policy makers’ circumstances. For example, given that dietary habits and levels of alcohol consumption in homeless populations in South Korea are likely to be different to those in Europe or North America, this study is unlikely to provide information relevant to European policy makers, and vice versa. There are variations in diets from one part of the world to another as well as changes in diets over time [36, 37]. Similar to diet variation across time and region, fortification practices have varied within and across regions in the past [38, 39]. While most participants in individual studies were from hostels or shelters, these may not represent the majority who do not access these services [40] and there were few studies which included rough sleepers. All these factors resulted in relatively sparse and unreliable evidence being available for interpretation and our findings are limited by this.

Furthermore, the measurement of vitamin deficiencies has apparently also evolved over time and the reference ranges vary with methods and techniques used, and across populations [41–44]. It was therefore difficult to interpret (and inappropriate to pool) data across studies. This field may benefit from an international initiative to reach expert consensus on standardized definitions and thresholds for nutritional deficiencies. This could lead to future studies that are more informative to policy makers. Similar initiatives to develop core outcome sets for clinical trials have led to subsequent trials assessing comparable and relevant outcomes and thus contributing more meaningfully to the overall body of evidence in their fields [45, 46].

The complications of nutritional deficiencies in homeless problem-drinkers can be avoided if deficiencies are addressed early. A practical intervention would be a fortified food or supplement distributed to the homeless heavy drinkers. Such an intervention would work best when tailored to specific deficiencies in the target group. Provision of vitamin rich packets of chocolate paste has been found acceptable to rough sleepers in Paris [47] but there was no assessment of any resulting change in nutritional status. However, the included Darnton-Hill study [13] showed that providing supplements can change deficiency levels in this population.

Until a tailored nutritional or other relevant intervention can be developed to provide a long term solution, existing evidence can be used to improve the short term outcomes relevant to a healthcare system and society and to the homeless drinkers who suffer from deficiencies. Provision of vitamin supplements may prove useful [13, 48]. Fortification of beer [22, 49–51] although tempting, constitutes an ethical dilemma of turning a ‘bad product’ in to a ‘super food’ [52]. The idea is however not very different from needle exchange programs – the final aim being the prevention of disease when the risky behaviour cannot always be stopped [53, 54].

The available evidence is not sufficient to inform the development of a public health intervention for homeless drinkers. For example, the findings do not tell us which nutrients are lacking (and should be targeted) nor to what extent these deficiencies exist in the homeless heavy drinkers in specific countries today. A carefully designed study including a representative population in a specific country would be more useful to inform the development of such an intervention. The available data from the included studies did not allow us to draw conclusions on the importance of social factors (such as poverty, mental health, or family breakdown) but we acknowledge that addressing these issues could be part of the solution.

A representative sample is essential when studying prevalence. Homeless people are not easy to reach however, [55] so it may be practical to carry out a registry-study of recent hospital/clinic records of the attending homeless problem-drinking patients. Such an analysis would be more representative than a small convenient sample.

## Conclusions

In summary, the limited, low quality, and relatively old evidence suggests that homeless problem-drinkers may be deficient in vitamin C, thiamine, and other nutrients to varying levels. However, this is not a complete picture. Further well conducted studies are needed to inform public health interventions to improve deficiencies. But any future studies will be more useful if they are informed by expert consensus on definitions and thresholds for nutritional deficiencies, leading to comparable outcome measures across studies. Studies carried out in one country or community may not necessarily be suitable for informing public health policy in a different setting due to the differences in access to food, shelter and services, levels of alcohol consumption or local food fortification practices.

## Additional files

Additional file 1: List of excluded studies. (PDF 338 kb)

Additional file 2: Graphic presentation of vitamin deficiencies in included studies. (JPG 579 kb)

## Abbreviations

A&E: Accident and emergency
B1: Thiamine
B12: Cobalamins
B2: Riboflavin
B3: Niacin
B5: Pantothenic acid
B6: Pyridoxine
B7: Biotin
B9: Folic acid
F: Female
Hb: Haemoglobin
HPLC: High performance liquid chromatography
IQR: Interquartile range
M: Male
NR: Not reported
P5P: Pyridoxal 5 phosphate
PLP: Pyridoxal 5 phosphate
TDP: Thiamine diphosphate
TK: Transketolase
TPP: Thiamine pyrophosphate
Vit A: Retinol/beta carotene
Vit C: Ascorbic acid
Vit D: Ergo/cholecalciferol
Vit E: Tocopherols
