# Supplementary material for: Nutritional deficiencies in homeless persons with problematic drinking: a systematic review
Source: Int J Equity Health. 2017 May 5;16:71. doi: 10.1186/s12939-017-0564-4 (PMC5418701; doi:10.1186/s12939-017-0564-4)
Supplement: Supplementary file 1 — List of excluded studies. (PDF 338 kb) [file 12939_2017_564_MOESM1_ESM.pdf]

Excluded papers with reasons (n= 195)

No homeless participants reported (n=79)

[1-78]

Problem drinkers few or none reported (n=29)

[79-107]

No information on blood or urine nutrient levels reported (n=29)

[108-136]

Not an empirical study (reviews/ comments) (n=57)

[137-193]

Deficiency due to TB medicine (n=1)

[194]

Bibliography:

1. Biery, J.R., J.H. Williford, Jr., and E.A. McMullen, *Alcohol craving in rehabilitation: assessment of nutrition therapy.* Journal of the American Dietetic Association, 1991. **91**(4): p. 463-6.
2. Kretschmar, C., S. Kaumeier, and W. Haase, *[Medicamentous therapy of alcoholic polyneuropathy. Randomized double-blind study comparing 2 vitamin B preparations and a nucleotide preparation].* Fortschritte der Medizin, 1996. **114**(32): p. 439-43.
3. Loguercio, C., et al., *Can dietary intake influence plasma levels of amino acids in liver cirrhosis?* Digestive & Liver Disease, 2000. **32**(7): p. 611-6.
4. Lopez, D.G., et al., *[Nutritional study of alcoholics with specific reference to calcium and phosphorus].* Revista Medica de Chile, 1991. **119**(6): p. 652-8.
5. Pinedo, S., et al., *[Protein-calorie malnutrition and degree of hepatic failure in chronic hepatopathy].* Revista Espanola de Enfermedades Digestivas, 1993. **84**(6): p. 381-5.
6. Pongpaev, P., et al., *Alcohol consumption, liver function tests and nutritional status in Thai males.* International Journal for Vitamin & Nutrition Research, 1981. **51**(4): p. 391-400.
7. Safa, G., et al., *[Survey in 2000: 3 cases].* Annales de Dermatologie et de Venereologie, 2001. **128**(11): p. 1225-8.
8. Tramacere, I., et al., *A meta-analysis on alcohol drinking and gastric cancer risk.* Annals of Oncology, 2012. **23**(1): p. 28-36.
9. Phillips, G.B., G.J. Gabuzda, Jr., and C.S. Davidson, *Comparative effects of a purified and an adequate diet on the course of fatty cirrhosis in the alcoholic.* Journal of Clinical Investigation, 1952. **31**(4): p. 351-6.
10. Baker, H., et al., *Inability of chronic alcoholics with liver disease to use food as a source of folates, thiamin and vitamin B6.* American Journal of Clinical Nutrition, 1975. **28**(12): p. 1377-80.
11. Sheremata, W.A. and I. Sherwin, *Alcoholic myelopathy. With spastic urinary bladder.* Diseases of the Nervous System, 1972. **33**(2): p. 136-9.
12. Lieber, C.S., *Alcohol-nutrition interaction.* Boletin - Asociacion Medica de Puerto Rico, 1984. **76**(10): p. 445-7.
13. Payne, I.R., G.H. Lu, and K. Meyer, *Relationship of dietary tryptophan and niacin to tryptophan metabolism in alcoholics and nonalcoholics.* American Journal of Clinical Nutrition, 1974. **27**(6): p. 572-9.
14. Barth, L., *[Treatment of acute incidents in drug addiction].* Nervenarzt, 1972. **43**(5): p. 266-7.
15. Forattini, C., *[Nucleodoxin in the treatment of alcoholic myocardiopathies. Hemodynamic evaluations with the polygraphic method].* Omnia Medica et Therapeutica, 1970. **48**(3): p. 361-73.
16. Eichner, E.R., N. Buergel, and R.S. Hillman, *Experience with an appetizing, high protein, low folate diet in man.* American Journal of Clinical Nutrition, 1971. **24**(11): p. 1337-45.
17. Labadarios, D., et al., *Vitamin B6 deficiency in chronic liver disease--evidence for increased degradation of pyridoxal-5'-phosphate.* Gut, 1977. **18**(1): p. 23-7.
18. Peters, R.S., A. Longtin, and J.D. Boyle, *Use of a diet supplement in Laennec's cirrhosis.* Gastroenterology, 1968. **54**(5): p. 872-5.
19. Kohlschutter, S., E. Lang, and H. Gofferje, *[Nutritional rehabilitation following chronic alcohol abuse].* MMW - Munchener Medizinische Wochenschrift, 1982. **124**(7): p. 163-5.
20. Woelk, H., et al., *Benfotiamine in treatment of alcoholic polyneuropathy: an 8-week randomized controlled study (BAP I Study).* Alcohol & Alcoholism, 1998. **33**(6): p. 631-8.
21. Guerrini, I. and R. Mundt-Leach, *Preventing long-term brain damage in alcohol-dependent patients.* Nursing Standard, 2013. **27**(19): p. 43-6.
22. Keal, E.E. and M. Harington, *Cirrhosis and hypoxia.* Proceedings of the Royal Society of Medicine, 1970. **63**(6): p. 621-2.
23. Gachnochi, G. and A. Soulairac, *[Clinical and therapeutic aspects of psychiatric alcoholism].* Semaine des Hopitaux, 1972. **48**(10): p. 697-703.

24. Bates, R.C., *Dietary treatment of alcoholism.* Modern Treatment, 1966. **3**(3): p. 556-60.
25. Iber, F.L., et al., *Vitamin K deficiency in chronic alcoholic males.* Alcoholism: Clinical & Experimental Research, 1986. **10**(6): p. 679-81.
26. Manzardo, A.M., et al., *Double-blind, randomized placebo-controlled clinical trial of benfotiamine for severe alcohol dependence.* Drug & Alcohol Dependence, 2013. **133**(2): p. 562-70.
27. Day, G.S. and C.M. del Campo, *Wernicke encephalopathy: a medical emergency.* CMAJ Canadian Medical Association Journal, 2014. **186**(8): p. E295.
28. Toniolo, P., E. Riboli, and A.P. Cappa, *A community study of alcohol consumption and dietary habits in middle-aged Italian women.* International Journal of Epidemiology, 1991. **20**(3): p. 663-70.
29. Labadarios, D., et al., *Pyridoxine deficiency in severe liver disease [proceedings].* Proceedings of the Nutrition Society, 1976. **35**(3): p. 141A.
30. Buydens-Branchey, L., M. Branchey, and J.R. Hibbeln, *Associations between increases in plasma n-3 polyunsaturated fatty acids following supplementation and decreases in anger and anxiety in substance abusers.* Progress in Neuro-Psychopharmacology & Biological Psychiatry, 2008. **32**(2): p. 568-75.
31. Videla, L.A., et al., *Content of hepatic reduced glutathione in chronic alcoholic patients: influence of the length of abstinence and liver necrosis.* Clinical Science, 1984. **66**(3): p. 283-90.
32. Jukic, T., et al., *The use of a food supplementation with D-phenylalanine, L-glutamine and L-5-hydroxytriptophan in the alleviation of alcohol withdrawal symptoms.* Collegium Antropologicum, 2011. **35**(4): p. 1225-30.
33. Kane, R.L., *Community medicine on the Navajo Reservation.* HSMHA Health Reports, 1971. **86**(8): p. 733-40.
34. Roberts, C.M., et al., *Malnutrition and a rash: think zinc.* Clinical & Experimental Dermatology, 2007. **32**(6): p. 654-7.
35. Piatkowski, J., P. Wiechert, and K. Ernst, *Ascorbic acid in chronic alcoholics.* International Journal for Vitamin & Nutrition Research, 1986. **56**(4): p. 421.
36. Barbadoro, P., et al., *Fish oil supplementation reduces cortisol basal levels and perceived stress: a randomized, placebo-controlled trial in abstinent alcoholics.* Molecular Nutrition & Food Research, 2013. **57**(6): p. 1110-4.
37. Kroenke, C.H., et al., *A cross-sectional study of alcohol consumption patterns and biologic markers of glycemic control among 459 women.* Diabetes Care, 2003. **26**(7): p. 1971-8.
38. Gonzalez-Reimers, E., et al., *Prognostic value of serum selenium levels in alcoholics.* Biological Trace Element Research, 2008. **125**(1): p. 22-9.
39. Baines, M., J.G. Bligh, and J.S. Madden, *Tissue thiamin levels of hospitalised alcoholics before and after oral or parenteral vitamins.* Alcohol & Alcoholism, 1988. **23**(1): p. 49-52.
40. Ng, T.M. and I.E. Bajjoka, *Treatment options for osteoporosis in chronic liver disease patients requiring liver transplantation.* Annals of Pharmacotherapy, 1999. **33**(2): p. 233-5.
41. Ambrose, M.L., S.C. Bowden, and G. Whelan, *Thiamin treatment and working memory function of alcohol-dependent people: preliminary findings.* Alcoholism: Clinical & Experimental Research, 2001. **25**(1): p. 112-6.
42. Kiefer, F., et al., *Leptin: a modulator of alcohol craving?* Biological Psychiatry, 2001. **49**(9): p. 782-7.
43. Ritson, B., *Treatment for alcohol related problems.* BMJ, 2005. **330**(7483): p. 139-41.
44. Sutamnartpong, P., S. Muengtaweepongsa, and K. Kulkantrakorn, *Wernicke's encephalopathy and central pontine myelinolysis in hyperemesis gravidarum.* Journal of Neurosciences in Rural Practice, 2013. **4**(1): p. 39-41.
45. Laven, G.T. and K.C. Brown, *Nutritional status of men attending a soup kitchen: a pilot study.* Am J Public Health, 1985. **75**(8): p. 875-8.

46. Forrester, J.E., et al., *Body composition and dietary intake in relation to drug abuse in a cohort of HIV-positive persons.* Journal of Acquired Immune Deficiency Syndromes: JAIDS, 2000. **25 Suppl 1**: p. S43-8.
47. Fox, R.P., M.B. Graham, and M.J. Gill, *A therapeutic revolving door.* Archives of General Psychiatry, 1972. **26**(2): p. 179-82.
48. Majumdar, S.K., et al., *Vitamin C utilization status in chronic alcoholic patients after short-term intravenous therapy.* International Journal for Vitamin & Nutrition Research, 1981. **51**(3): p. 274-8.
49. Sherlock, S., *Nutrition and the alcoholic.* Lancet, 1984. **1**(8374): p. 436-9.
50. Resnick, R.H. and F.L. Iber, *Treatment of acute alcoholic hepatitis.* Gut, 1972. **13**(1): p. 68-73.
51. Kuffner, E.K., et al., *The effect of acetaminophen (four grams a day for three consecutive days) on hepatic tests in alcoholic patients--a multicenter randomized study.* BMC Medicine, 2007. **5**: p. 13.
52. Grimble, G.K., *Current international literature citation--nutrition.[Erratum appears in Nutrition 1999 Oct;15(10):739].* Nutrition, 1999. **15**(7-8): p. 646-7.
53. Phillips, G.B. and C.S. Davidson, *Nutritional aspects of cirrhosis in alcoholism; effect of a purified diet supplemented with choline.* Annals of the New York Academy of Sciences, 1954. **57**(6): p. 812-30.
54. Bianchi, A., et al., *Nutritional folic acid deficiency with megaloblastic changes in the small-bowel epithelium.* New England Journal of Medicine, 1970. **282**(15): p. 859-61.
55. Barbadoro, P., et al., *The effects of educational intervention on nutritional behaviour in alcohol-dependent patients.* Alcohol & Alcoholism, 2011. **46**(1): p. 77-9.
56. LoVecchio, F., *Multivitamin "banana bags" provide little value in emergency department patients.* Annals of Emergency Medicine, 2012. **59**(5): p. 414-5.
57. Majumdar, S.K., G.K. Shaw, and A.D. Thomson, *Blood vitamin status in chronic alcoholics after a single dose of polyvitamin. A preliminary report.* Postgraduate Medical Journal, 1981. **57**(665): p. 164-6.
58. Centerwall, B.S. and M.H. Criqui, *Prevention of the Wernicke-Korsakoff syndrome: a cost-benefit analysis.* New England Journal of Medicine, 1978. **299**(6): p. 285-9.
59. Cole, M., et al., *Extraocular palsy and thiamine therapy in Wernicke's encephalopathy.* American Journal of Clinical Nutrition, 1969. **22**(1): p. 44-51.
60. Somogyi, J.C. and P.M. Kopp, *Relation between chronic alcoholism, drug addiction and nutrition with special reference to the thiamine status.* Bibliotheca Nutritio et Dieta, 1981(30): p. 131-8.
61. Naidoo, D.P., A. Bramdev, and K. Cooper, *Wernicke's encephalopathy and alcohol-related disease.* Postgraduate Medical Journal, 1991. **67**(793): p. 978-81.
62. Wood, B. and J. Currie, *Presentation of acute Wernicke's encephalopathy and treatment with thiamine.* Metabolic Brain Disease, 1995. **10**(1): p. 57-72.
63. Atkinson, G.W. and W.C. Kappes, Jr., *Pyridoxine (vitamin B6) in alcoholism.* Virginia Medical Monthly, 1956. **83**(9): p. 391-3.
64. Marques, A.C.P.R. and E.F. Furtado, *Brief interventions for alcohol related problems. [Portuguese].* Revista Brasileira de Psiquiatria, 2004. **26**(SUPPL.): p. 28-32.
65. Bjelakovic, G., et al., *Vitamin D supplementation for chronic liver disease-a cochrane hepato-biliary group systematic review.* Journal of Hepatology, 2015. **62**: p. S837.
66. Riserus, U. and E. Ingelsson, *Alcohol intake, insulin resistance, and abdominal obesity in elderly men.* Obesity, 2007. **15**(7): p. 1766-73.
67. Overall, J.E., et al., *Drug treatment of anxiety and depression in detoxified alcoholic patients.* Archives of General Psychiatry, 1973. **29**(2): p. 218-25.
68. Majumdar, S.K., G.K. Shaw, and A.D. Thomson, *Plasma vitamin E status in chronic alcoholic patients.* Drug & Alcohol Dependence, 1983. **12**(3): p. 269-72.

69. dos Santos, J.E., et al., *Nutritional care of hospitalized patients in Brazil with particular reference to pellagra and alcoholism as complicating factors.* Progress in Clinical & Biological Research, 1981. **77**: p. 719-27.
70. Sobral-Oliveira, M.B., et al., *Nutritional profile of asymptomatic alcoholic patients.* Arquivos de Gastroenterologia, 2011. **48**(2): p. 112-8.
71. Smith, J.A., P.A. Dardin, and W.T. Brown, *The treatment of alcoholism by nutritional supplement.* Quarterly Journal of Studies on Alcohol, 1951. **12**(3): p. 381-5.
72. Rees, E. and L.R. Gowing, *Supplementary thiamine is still important in alcohol dependence.* Alcohol & Alcoholism, 2013. **48**(1): p. 88-92.
73. Losowsky, M.S. and P.J. Leonard, *Evidence of vitamin E deficiency in patients with malabsorption or alcoholism and the effects of therapy.* Gut, 1967. **8**(6): p. 539-43.
74. Lindenbaum, J. and C.S. Lieber, *Hematologic effects of alcohol in man in the absence of nutritional deficiency.* New England Journal of Medicine, 1969. **281**(7): p. 333-8.
75. Iber, F.L., *Evaluation of an oral solution to accelerate alcoholism detoxification.* Alcoholism: Clinical & Experimental Research, 1987. **11**(3): p. 305-8.
76. Tang, A.M., et al., *Malnutrition in a population of HIV-positive and HIV-negative drug users living in Chennai, South India.* Drug & Alcohol Dependence, 2011. **118**(1): p. 73-7.
77. Wilkens Knudsen, A., et al., *Nutritional intake and status in persons with alcohol dependency: data from an outpatient treatment programme.* European Journal of Nutrition, 2014. **53**(7): p. 1483-92.
78. Trulson, M.F., R. Fleming, and F.J. Stare, *Vitamin medication in alcoholism.* Journal of the American Medical Association, 1954. **155**(2): p. 114-9.
79. Antoniades, M. and V. Tarasuk, *A survey of food problems experienced by Toronto street youth.* Canadian Journal of Public Health. Revue Canadienne de Sante Publique, 1998. **89**(6): p. 371-5.
80. Bunston, T. and M. Breton, *The eating patterns and problems of homeless women.* Women & Health, 1990. **16**(1): p. 43-62.
81. Dressler, H. and C. Smith, *Health and eating behavior differs between lean/normal and overweight/obese low-income women living in food-insecure environments.* American Journal of Health Promotion, 2013. **27**(6): p. 358-65.
82. Koh, K.A., et al., *The hunger-obesity paradox: obesity in the homeless.* Journal of Urban Health, 2012. **89**(6): p. 952-64.
83. Press, V.N.H.F.F.o.P.H., *Nutrition and Food Poverty: a toolkit for those involved in developing or implementing a local nutrition and food poverty strategy*. 2004, National Heart Forum: London. p. 163pp.
84. Luder, E., et al., *Assessment of the nutritional status of urban homeless adults.* Public Health Reports, 1989. **104**(5): p. 451-7.
85. Dachner, N., et al., *An ethnographic study of meal programs for homeless and under-housed individuals in Toronto.* Journal of Health Care for the Poor & Underserved, 2009. **20**(3): p. 846-53.
86. Austin, C.K., C.E. Goodman, and L.L. Van Halderen, *Absence of malnutrition in a population of homeless veterans.* Journal of the American Dietetic Association, 1996. **96**(12): p. 1283-5.
87. Taylor, M.L. and S.A. Koblinsky, *Dietary intake and growth status of young homeless children.* Journal of the American Dietetic Association, 1993. **93**(4): p. 464-6.
88. Stitt, S., A.M. Coufopoulos, and D. Grant, *HOMELESSNESS AND FOOD CHOICE - LET THEM EAT CAKE.* Appetite, 1995. **24**(3): p. 290-290.
89. Gelder, A.H.J., *Struggling to eat well: homelessness and healthy eating*. 2004, Housing Justice: London. p. 6pp.
90. Mejean, C., et al., *Associations of socioeconomic factors with inadequate dietary intake in food aid users in France (The ABENA study 2004-2005).[Erratum appears in Eur J Clin Nutr.*

2010 Apr;64(4):440 Note: Serge, H [corrected to Hercberg, S]; Katia, C [corrected to Castetbon, K]]. European Journal of Clinical Nutrition, 2010. **64**(4): p. 374-82.
91. Pelham-Burn, S.E., et al., *Improving the nutritional quality of charitable meals for homeless and vulnerable adults. A case study of food provision by a food aid organisation in the UK.* Appetite, 2014. **82**: p. 131-7.
92. Johnson, L.J., et al., *Nutrition education for homeless women - challenges and opportunities: a pilot study.* Journal of Foodservice Business Research, 2009. **12**(2): p. 155-169.
93. Hamm, L.A. and E.W. Holden, *Providing WIC services to homeless families.* Journal of Nutrition Education, 1999. **31**(4): p. 224-229.
94. Fontaine, K.R., et al., *Body mass index and effects of refeeding on liver tests in drug-dependent adults in a residential research unit.* Journal of the American Dietetic Association, 2001. **101**(12): p. 1467-9.
95. Luder, E., et al., *Health and nutrition survey in a group of urban homeless adults.* Journal of the American Dietetic Association, 1990. **90**(10): p. 1387-92.
96. Wilson, A., N. Szwed, and A. Renzaho, *Developing Nutrition Guidelines for Recycled Food to Improve Food Security Among Homeless, Asylum Seekers, and Refugees in Victoria, Australia.* Journal of Hunger and Environmental Nutrition, 2012. **7**(2-3): p. 239-252.
97. Rascoe, D. and S. Dalton, *Hungry, homeless, and HIV: a study of homeless visitors to an outreach meal center for people with AIDS.* Journal of Nutrition Education, 1993. **25**(4): p. 205-207.
98. Visvanathan, R., et al., *The nutritional status of 1081 elderly people residing in publicly funded shelter homes in Peninsular Malaysia.* European Journal of Clinical Nutrition, 2005. **59**(3): p. 318-24.
99. Bell, M., L. Wilbur, and C. Smith, *Nutritional status of persons using a local emergency food system program in middle America.* Journal of the American Dietetic Association, 1998. **98**(9): p. 1031-1033.
100. Lyles, C.R., et al., *Nutritional assessment of free meal programs in San Francisco.* Preventing Chronic Disease, 2013. **10**: p. E90.
101. Linares, E., *Food services for the homeless in Spain: Caritas Programme for the Homeless.* Public Health Nutrition, 2001. **4**(6A): p. 1367-9.
102. Tse, C. and V. Tarasuk, *Nutritional assessment of charitable meal programmes serving homeless people in Toronto.* Public Health Nutrition, 2008. **11**(12): p. 1296-305.
103. Lowen, J.T., *A hot meal and a health check.* Minnesota Medicine, 2009. **92**(11): p. 8-9.
104. Drake, M.A., *The nutritional status and dietary adequacy of single homeless women and their children in shelters.* Public Health Reports, 1992. **107**(3): p. 312-9.
105. Point, C., *Toxic mix: the health needs of homeless young people*. 2014, Ccentre Point: London. p. 18pp.
106. Ober, K., L. Carlson, and P. Anderson, *Cardiovascular risk factors in homeless adults.* Journal of Cardiovascular Nursing, 1997. **11**(4): p. 50-9.
107. Sisson, L.G. and D.A. Lown, *Do soup kitchen meals contribute to suboptimal nutrient intake & obesity in the homeless population?* Journal of Hunger and Environmental Nutrition, 2011. **6**(3): p. 312-323.
108. Veenstra, J., et al., *Alcohol consumption in relation to food intake and smoking habits in the Dutch National Food Consumption Survey.* European Journal of Clinical Nutrition, 1993. **47**(7): p. 482-9.
109. Wolgemuth, J.C., et al., *Wasting malnutrition and inadequate nutrient intakes identified in a multiethnic homeless population.* Journal of the American Dietetic Association, 1992. **92**(7): p. 834-9.
110. Evans, N.S. and E.A. Dowler, *Food, health and eating among single homeless and marginalized people in London.* Journal of Human Nutrition and Dietetics, 1999. **12**(3): p. 179-199.

111. Hoher, A., *[Treatment of acute abstinence symptoms in chronic alcoholics].* Ugeskrift for Laeger, 1962. **124**: p. 506-9.
112. Derrickson, J.P., et al., *Lessons learned from the "Spend Less. Eat Well. Feel Better." program efficacy trial.* Journal of Nutrition Education & Behavior, 2003. **35**(1): p. 30-6.
113. Forrester, J.E., K.L. Tucker, and S.L. Gorbach, *Dietary intake and body mass index in HIV-positive and HIV-negative drug abusers of Hispanic ethnicity.* Public Health Nutrition, 2004. **7**(7): p. 863-70.
114. Kinder, H., *Implementing nutrition guidelines that will benefit homeless people.* Nursing Times, 2004. **100**(24): p. 32-4.
115. Gelberg, L. and L.S. Linn, *Social and physical health of homeless adults previously treated for mental health problems.* Hospital & Community Psychiatry, 1988. **39**(5): p. 510-516.
116. Hickey, C.D., D.;Focus Ireland, *Hungry for change: social exclusion,food poverty and homelessness in Dublin: a pilot research study*. 2003, Focus Ireland: Dublin. p. 133pp.
117. Silfverskiold, B.P., *[The Wernicke-Korsakoff syndrome and ataxia in alcoholics].* Lakartidningen, 1969. **66**(46): p. 4773-8.
118. Carillo, T.E., J.A. Gilbride, and M.M. Chan, *Soup kitchen meals: an observation and nutrient analysis.* Journal of the American Dietetic Association, 1990. **90**(7): p. 989-91.
119. Fain, O., *[Vitamin C deficiency].* Revue de Medecine Interne, 2004. **25**(12): p. 872-80.
120. Link, H., *The unhealthy state of homelessness*. 2014, Homeless Link: London. p. 23pp.
121. CHNI, *Nutrition for alcohol users: a resource booklet for staff working with homeless alcohol users*, CHNI, Editor. 2013, CHNI: Belfast. p. 20pp.
122. Wicks, R., L.J. Trevena, and S. Quine, *Experiences of food insecurity among urban soup kitchen consumers: insights for improving nutrition and well-being.* Journal of the American Dietetic Association, 2006. **106**(6): p. 921-4.
123. Bennett, J.B. and A. Scholler-Jaquish, *The winner's group: a self-help group for homeless chemically dependent persons.* Journal of Psychosocial Nursing & Mental Health Services, 1995. **33**(4): p. 14-9.
124. Normen, L., et al., *Food insecurity and hunger are prevalent among HIV-positive individuals in British Columbia, Canada.* Journal of Nutrition, 2005. **135**(4): p. 820-5.
125. Bowering, J., K.L. Clancy, and J. Poppendieck, *Characteristics of a random sample of emergency food program users in New York: II. Soup kitchens.* American Journal of Public Health, 1991. **81**(7): p. 914-7.
126. Farkas, M.E. and J. Dwyer, *Nutrition education for alcoholic recovery homes.* Journal of Nutrition Education, 1984. **16**(3): p. 123-124.
127. Tsai, J. and R.A. Rosenheck, *Obesity among chronically homeless adults: is it a problem?* Public Health Reports, 2013. **128**(1): p. 29-36.
128. Nowson, C.A., et al., *Energy, protein, calcium, vitamin D and fibre intakes from meals in residential care establishments in Australia.* Asia Pacific Journal of Clinical Nutrition, 2003. **12**(2): p. 172-7.
129. Darmon, N., *A fortified street food to prevent nutritional deficiencies in homeless men in France.* Journal of the American College of Nutrition, 2009. **28**(2): p. 196-202.
130. Crowley, A.D.U., *Making it matter: improving the health of young homeless people*. 2012, Depaul UK: London. p. 72pp.
131. Agency, D.M.L.F.S., *Research into Food Poverty and homelessness in Northern Ireland: Final report*. 2006, Food Standards Agency: Belfast. p. 93pp.
132. Darmon, N., et al., *Dietary inadequacies observed in homeless men visiting an emergency night shelter in Paris.* Public Health Nutrition, 2001. **4**(2): p. 155-61.
133. Baggett, T.P., et al., *Food insufficiency and health services utilization in a national sample of homeless adults.* Journal of General Internal Medicine, 2011. **26**(6): p. 627-34.

134. Wiecha, J.L., et al., *Nutritional and economic advantages for homeless families in shelters providing kitchen facilities and food.* Journal of the American Dietetic Association, 1993. **93**(7): p. 777-83.
135. Rustad, C. and C. Smith, *Nutrition knowledge and associated behavior changes in a holistic, short-term nutrition education intervention with low-income women.* Journal of Nutrition Education & Behavior, 2013. **45**(6): p. 490-8.
136. Arthur SG., A.F.H.a.S.M.M., *Proceedings of the Nutrition Society: abstracts of original communications... A scientific meeting was held at the King's College, London, UK on 7-10 July 2003.* Proceedings of the Nutrition Society, 2003. **62**: p. 35A-93a.
137. Anonymous, *Influence of malnutrition and alcohol on thiamine absorption.* Nutrition Reviews, 1971. **29**(1): p. 13-5.
138. Bikle, D.D., *Alcohol-induced bone disease.* World Review of Nutrition & Dietetics, 1993. **73**: p. 53-79.
139. Binns, C.W., S.J. Carruthers, and P.A. Howat, *Thiamin in beer: a health promotion perspective.* Community Health Studies, 1989. **13**(3): p. 301-5.
140. Bishai, D.M. and L.P. Bozzetti, *Current progress toward the prevention of the Wernicke-Korsakoff syndrome.* Alcohol & Alcoholism, 1986. **21**(4): p. 315-23.
141. Bleich, S., K. Loffelholz, and J. Kornhuber, *[Folate against hyperhomocysteinemia. A new approach for the prevention and therapy of alcoholism-associated disorders?].* Nervenarzt, 2004. **75**(5): p. 425-30.
142. Cambier, J., R. Dairou, and M. Gonce, *[Gayet-Wernicke encephalopathy].* Journal of the Royal College of Physicians of London, 1977. **12**(1): p. 21-9.
143. Clifford, P.R. and S.A. Maisto, *Subject reactivity effects and alcohol treatment outcome research.* Journal of Studies on Alcohol, 2000. **61**(6): p. 787-93.
144. Cook, C.C., P.M. Hallwood, and A.D. Thomson, *B Vitamin deficiency and neuropsychiatric syndromes in alcohol misuse.* Alcohol & Alcoholism, 1998. **33**(4): p. 317-36.
145. Cylwik, B. and L. Chrostek, *[Disturbances of folic acid and homocysteine metabolism in alcohol abuse].* Polski Merkuriusz Lekarski, 2011. **30**(178): p. 295-9.
146. Galambos, J.T., *Alcoholic hepatitis: its therapy and prognosis.* Progress in Liver Diseases, 1972. **4**: p. 567-88.
147. Gray, G.E., *Nutrition and dementia.* Journal of the American Dietetic Association, 1989. **89**(12): p. 1795-802.
148. Hinze-Selch, D., et al., *[Thiamine treatment in psychiatry and neurology].* Fortschritte der Neurologie-Psychiatrie, 2000. **68**(3): p. 113-20.
149. Leone, D., et al., *[Drug/food interactions: an actual therapeutic outcome].* Clinica Terapeutica, 2004. **155**(4): p. 139-47.
150. Lieber, C.S., *Relationships between nutrition, alcohol use, and liver disease.* Alcohol Research & Health: the Journal of the National Institute on Alcohol Abuse & Alcoholism, 2003. **27**(3): p. 220-31.
151. Lutz, U.C., *Alterations in homocysteine metabolism among alcohol dependent patients--clinical, pathobiochemical and genetic aspects.* Current Drug Abuse Reviews, 2008. **1**(1): p. 47-55.
152. Meier, S. and J.B. Daeppen, *[Prevalence, prophylaxis and treatment of Wernicke encephalopathy. Thiamine, how much and how do we give it?].* Revue Medicale Suisse, 2005. **1**(26): p. 1740-4.
153. Roe, D.A., *Nutritional concerns in the alcoholic.* Journal of the American Dietetic Association, 1981. **78**(1): p. 17-21.
154. Salaspuro, M., *Nutrient intake and nutritional status in alcoholics.* Alcohol & Alcoholism, 1993. **28**(1): p. 85-8.

155. Thomson, A.D., *Mechanisms of vitamin deficiency in chronic alcohol misusers and the development of the Wernicke-Korsakoff syndrome.* Alcohol & Alcoholism. Supplement, 2000. **35**(1): p. 2-7.
156. Thomson, A.D. and C.C. Cook, *Parenteral thiamine and Wernicke's encephalopathy: the balance of risks and perception of concern.* Alcohol & Alcoholism, 1997. **32**(3): p. 207-9.
157. Thomson, A.D., et al., *The Royal College of Physicians report on alcohol: guidelines for managing Wernicke's encephalopathy in the accident and Emergency Department.[Erratum appears in Alcohol Alcohol. 2003 May-Jun;38(3):291].* Alcohol & Alcoholism, 2002. **37**(6): p. 513-21.
158. Thomson, A.D., et al., *Thiamine propyl disulfide: absorption and utilization.* Annals of Internal Medicine, 1971. **74**(4): p. 529-34.
159. Yeomans, M.R. and R.W. Gray, *Opioid peptides and the control of human ingestive behaviour.* Neuroscience & Biobehavioral Reviews, 2002. **26**(6): p. 713-28.
160. Kokavec, A., *Is decreased appetite for food a physiological consequence of alcohol consumption?* Appetite, 2008. **51**(2): p. 233-43.
161. Horton, L., et al., *Comprehensive assessment of alcohol-related brain damage (ARBD): gap or chasm in the evidence?* Journal of Psychiatric & Mental Health Nursing, 2015. **22**(1): p. 3-14.
162. Achord, J.L., *Malnutrition and the role of nutritional support in alcoholic liver disease.* American Journal of Gastroenterology, 1987. **82**(1): p. 1-7.
163. Davis, R.E., *Clinical chemistry of vitamin B12.* Advances in Clinical Chemistry, 1985. **24**: p. 163-216.
164. Wiecha, J.L., J.T. Dwyer, and M. Dunn-Strohecker, *Nutrition and health services needs among the homeless.* Public Health Reports, 1991. **106**(4): p. 364-74.
165. Gronroos, N.N. and A. Alonso, *Diet and risk of atrial fibrillation - epidemiologic and clinical evidence.* Circulation Journal, 2010. **74**(10): p. 2029-38.
166. Bonjour, J.P., *Vitamins and alcoholism. II. folate and vitamin B12.* International Journal for Vitamin & Nutrition Research, 1980. **50**(1): p. 96-121.
167. Somogyi, J.C., *Early signs of thiamine deficiency.* Bibliotheca Nutritio et Dieta, 1976(23): p. 78-85.
168. Casacchia, M., *[The physician face to face with the alcohol-abusing patient].* Clinica Terapeutica, 1984. **109**(3): p. 277-82.
169. Katz, K.D., *Intravenous multivitamins ("banana bags") for emergency patients who may have nutritional deficits.* Annals of Emergency Medicine, 2012. **59**(5): p. 413-4.
170. Flink, E.B., *Magnesium deficiency.* Rocky Mountain Medical Journal, 1974. **71**(7): p. 396-7.
171. Prasad, A.S., *Clinical manifestations of zinc deficiency.* Annual Review of Nutrition, 1985. **5**: p. 341-63.
172. Hamm, S.S.H.C., *Homeless Nutrition Education Toolkit: a resource for nutrition educators and food providers*. 2012, Sacramento Hunger Coalition: Sacramento. p. 94pp.
173. Lindenbaum, J., *Folate and vitamin B12 deficiencies in alcoholism.* Seminars in Hematology, 1980. **17**(2): p. 119-29.
174. Morgan, M.Y., *Alcohol and nutrition.* British Medical Bulletin, 1982. **38**(1): p. 21-9.
175. Ogershok, P.R., et al., *Wernicke encephalopathy in nonalcoholic patients.* American Journal of the Medical Sciences, 2002. **323**(2): p. 107-11.
176. Halsted, C.H., *Alcoholism and malnutrition. Introduction to the symposium.* American Journal of Clinical Nutrition, 1980. **33**(12): p. 2705-8.
177. Sher, L., *Role of selenium depletion in the etiopathogenesis of depression in patients with alcoholism [corrected].[Erratum appears in Med Hypotheses. 2003 Sep;61(3):416].* Medical Hypotheses, 2002. **59**(3): p. 330-3.
178. Harper, C., *Thiamine (vitamin B1) deficiency and associated brain damage is still common throughout the world and prevention is simple and safe!* European Journal of Neurology, 2006. **13**(10): p. 1078-82.

179. Mitchell, M.C. and H.F. Herlong, *Alcohol and nutrition: caloric value, bioenergetics, and relationship to liver damage.* Annual Review of Nutrition, 1986. **6**: p. 457-74.
180. Leevy, C.M., *Thiamin deficiency and alcoholism.* Annals of the New York Academy of Sciences, 1982. **378**: p. 316-26.
181. D'Orazio, L. and P. D'Orazio, *Nutritional polyneuropathy and its manifestations in the lower extremity.* Journal of the American Podiatric Medical Association, 1985. **75**(1): p. 28-30.
182. Mezey, E., *Alcoholic liver disease: roles of alcohol and malnutrition.* American Journal of Clinical Nutrition, 1980. **33**(12): p. 2709-18.
183. Anonymous, *VITAMIN treatment of alcoholism.* Nutrition Reviews, 1954. **12**(7): p. 202-3.
184. Scott, M., *The dietetic side of liver disease management.* Dimensions in Health Service, 1975. **52**(8): p. 18, 40.
185. Baden, E., *Prevention of cancer of the oral cavity and pharynx.* CA: a Cancer Journal for Clinicians, 1987. **37**(1): p. 49-62.
186. Fuchs, J., *Alcoholism, malnutrition, vitamin deficiencies, and the skin.* Clinics in Dermatology, 1999. **17**(4): p. 457-461.
187. Ritson, E.B., *Psychological medicine. Treatment of alcoholism.* British Medical Journal, 1975. **2**(5963): p. 124-7.
188. Bjorneboe, A., G.E. Bjorneboe, and C.A. Drevon, *Absorption, transport and distribution of vitamin E.* Journal of Nutrition, 1990. **120**(3): p. 233-42.
189. Strasser, J.A., S. Damrosch, and J. Gaines, *Nutrition and the homeless person.* Journal of Community Health Nursing, 1991. **8**(2): p. 65-73.
190. Carey, G.B., *Nutrition and alcoholism: problems and therapies.* Occupational Medicine, 1989. **4**(2): p. 311-26.
191. McNichol, R.W. and A.Y. Hoshino, *Therapy for deliria.* Current Psychiatric Therapies, 1975. **15**: p. 181-93.
192. Leevy, C.M., C. Tamburro, and F. Smith, *Alcoholism, drug addiction, and nutrition.* Medical Clinics of North America, 1970. **54**(6): p. 1567-75.
193. Wood, B., *Thiamin status in Australia.* World Review of Nutrition & Dietetics, 1985. **46**: p. 148-218.
194. Raza, Y. and Y. Averbukh, *Presentation of zinc deficiency as a perianal ulcerative rash in an alcoholic man.* Journal of General Internal Medicine, 2013. **28**: p. S375.
