# Supplementary figures and images for: Nutritional deficiencies in homeless persons with problematic drinking: a systematic review
Source: Int J Equity Health. 2017 May 5;16:71. doi: 10.1186/s12939-017-0564-4 (PMC5418701; doi:10.1186/s12939-017-0564-4)

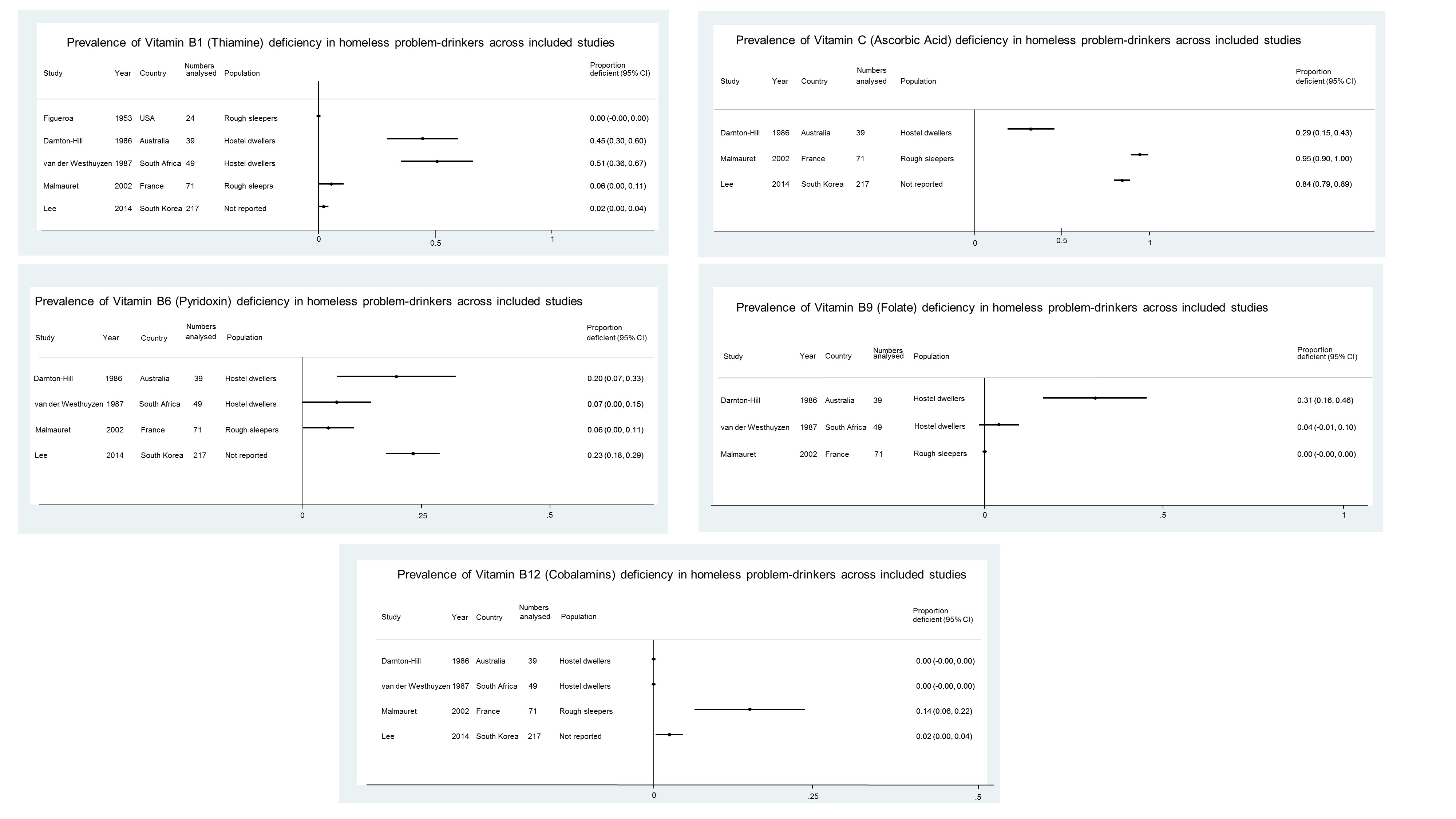

Supplement: Supplementary file 2 — Graphic presentation of vitamin deficiencies in included studies. (JPG 579 kb) [file 12939_2017_564_MOESM2_ESM.jpg]
